# Clinical Holistic Medicine (Mindful Short-Term Psychodynamic Psychotherapy Complimented with Bodywork) in the Treatment of Schizophrenia (ICD10-F20/DSM-IV Code 295) and Other Psychotic Mental Diseases

**DOI:** 10.1100/tsw.2007.298

**Published:** 2007-12-18

**Authors:** Søren Ventegodt, Isack Kandel, Joav Merrick

**Affiliations:** ^1^ Quality of Life Research Center, Teglgårdstræde 4-8, DK-1452 Copenhagen K, Denmark; ^2^ Research Clinic for Holistic Medicine, Copenhagen, Denmark; ^3^ Nordic School of Holistic Medicine, Copenhagen, Denmark; ^4^ Scandinavian Foundation for Holistic Medicine, Sandvika, Norway; ^5^ Interuniversity College, Graz, Austria; ^6^ Faculty of Social Sciences, Department of Behavioral Sciences, Ariel University Center, Samaria, Ariel, Israel; ^7^ National Institute of Child Health and Human Development, Jerusalem, Israel; ^8^ Office of the Medical Director, Division for Mental Retardation, Ministry of Social Affairs, Jerusalem, Israel; ^9^ Kentucky Children's Hospital, University of Kentucky, Lexington, USA

**Keywords:** mental health, psychiatry, holistic health and medicine

## Abstract

Clinical holistic medicine (CHM) has developed into a system that can also be helpful with mentally ill patients. CHM therapy supports the patient through a series of emotionally challenging, existential, and healing crises. The patient’s sense of coherence and mental health can be recovered through the process of feeling old repressed emotions, understanding life and self, and finally letting go of negative beliefs and delusions. The Bleuler's triple condition of autism, disturbed thoughts, and disturbed emotions that characterizes the schizophrenic patient can be understood as arising from the early defense of splitting, caused by negative learning from painful childhood traumas that made the patient lose sense of coherence and withdraw from social contact. Self-insight gained through the therapy can allow the patients to take their bodily, mental, and spiritual talents into use. At the end of therapy, the patients are once again living a life of quality centered on their life mission and they relate to other people in a way that systematically creates value. There are a number of challenges meeting the therapist who works with schizophrenic and psychotic patients, from the potential risk of experiencing a patient's violence, to the obligation to contain the most difficult and embarrassing of feelings when the emotional and often also sexual content of the patient’s unconsciousness becomes explicit. There is a long, well-established tradition for treating schizophrenia with psychodynamic therapy, and we have found that the combination of bodywork and psychotherapy can enhance and accelerate the therapy and might improve the treatment rate further.

## INTRODUCTION

Madness has tormented mankind since its birth and definitely as long back as we have medical recordings. Hippocrates (460–377 BCE) devoted attention to mental diseases and used an ingenuous combination of conversational therapy and bodywork as treatment for examples of hysteria (from Greek Hystera: Uterus) and other mental conditions[1]. As one of his treatments, he used pelvic massage[2,3] to “correct the energy of the uterus”, but also other provocative and efficient methods of interventions. To avoid the problems of sexual abuse following the use of bodywork and intimacy, he invented his famous Hippocratic ethics[1].

Philippe Pinel (1745–1826)[4] at Bicétre in Paris used “moral treatment” for mentally ill patients and he was surprisingly successful. According to his statistics, about 70% of the patients were healed using holistic medical principles. With a strong focus on medical ethics, he stressed the respect of the individual and used intensive studies of each single patient through detailed case recordings[4,5]. Pinel called his psychiatric therapy “traitement moral” and this holistic system represented the first modern attempt at individual psychotherapy. His treatment core values were gentleness, understanding, and goodwill; he was opposed to violent methods. He recommended close medical attendance during convalescence, and he emphasized the need of hygiene, physical exercise, and a program of purposeful work for the patient. A number of his therapeutic procedures, including ergo therapy and the placement of the patient in a family group, anticipated modern psychiatric care.

The Swiss psychiatrist Paul Eugen Bleuler (1857–1939) gave birth to the name schizophrenia (earlier named dementia praecox, but changed to characterize split mind in Greek) to describe the splitting in the mental functions between the patient and reality[5]. He had three main criterions, or primary symptoms, still being used in today’s psychiatry for diagnosing schizophrenia:

- Disturbed thought, especially difficulties in the logic and disorganization of thoughts
- Disturbed emotions, especially the presence of irrelevant feelings and numbness
- Autism, i.e., social withdrawal

This triad was complemented by a number of secondary symptoms, such as hallucinations, fixed delusions, catatonia, lack of self-care, and strange ways of speaking and acting. During the last century, the schizophrenic state was understood in more and more depth psychodynamically, and was seen as caused by the mental defense called “splitting”, during which a substantial part of the patient’s consciousness, emotional and sexual life is repressed[6,7]; splitting is a different, more primitive, and more radical defense than what we usually call repression in therapy. Spitting was understood psychodynamically as being the emotionally challenged child’s psychic survival position. The purpose of assuming the position of splitting was saving the patient’s vulnerable core of existence from the threat of destruction by the insensitive contact with parents not meeting the child’s sensitive mind and spirit the way it needed to be met. This is not to blame the parents who undoubtedly are doing their best, but some children are extremely sensitive and some parents are not.

This transformation of an open and vulnerable child into a protective person living in a safe shell of numbness and isolation explained how and why the patient turned herself into a disconnected and severely dysfunctional being. “Fear of dying” (or even panic) is, according to the psychodynamic theory, the permanent, emotional state of the schizophrenic patient. To cure the schizophrenic patient, the therapist must thus offer a “saving” that is perceived as safer than what is offered by the psychotic defenses. When the therapist is offering this alternative “safe place”, the patient can finally heal by opening up emotionally for the world once again, thus coming back to life and social life. There might, of course, be a constitutional factor on the side of the child, like what have been called “karmic traumas” (energetically inherited existential imbalances) that makes such a healing very difficult. Sigmund Freud (1856–1939)[6] and Carl Gustav Jung (1875–1961)[7] saw correctly the psychotic defenses as a very early mechanism (happening the first year of life or so or even before then, in the womb) and they were quite pessimistic about addressing this defense in therapy, as it firmly protected the fundamental existence of the being. Jung worked with Bleuler and was more optimistic than Freud in treating psychosis. The neurotic defense mechanisms like repression were seen as a more mature defense mechanism happening later in childhood, and only protecting the content of consciousness, not the container (the existence) itself. Freud believed that only the latter were curable by psychoanalysis. Psychodynamic psychotherapists like Harold Frederic Searles (1918–) found, in the middle of the last century, that the deep existential problems of preventing the loss of self through splitting were actually also curable by psychodynamic psychotherapy, and Searles became famous for curing about 33% of his schizophrenic patients this way; and many more great psychotherapists have been able to cure schizophrenia during the last century (see below under Discussion).

Also today, psychiatric disorders can be treated by psychodynamic psychotherapy; this kind of therapy has recently been found in meta-analysis to be superior to standard psychiatric treatment[8–10]. Standard psychiatric drug treatment is not curing the patient (and is not claiming to do so either) and it has severe side effects/adverse effects from the psychopharmacological drugs and from negative implanted philosophy[11], making this treatment a less-than-perfect medical procedure[12]. However, it may still be useful in many cases, especially when psychotherapy has failed to help.

Unfortunately, psychotherapy with schizophrenic patients can be extremely challenging for the therapist, and medical treatment with neuroleptic drugs therefore seems more attractive and less time consuming than psychodynamic psychotherapy, holistic therapy, and other kinds of psychotherapy that induce recovery and salutogenesis. Only further research can tell us the fraction of patients that can be completely healed; even the most talented of therapists, such as Searles and Laing, had to admit their failure in their attempt to cure schizophrenic patients. There have been critiques of research with psychotherapy for schizophrenia because the research was mostly done without control groups, and with schizophrenic patients, we all know the poor outcome for the patients if not treated or if treated with biomedicine (antipsychotic drugs like chlorpromazine[12]), which is exactly why the therapists did not bother to have a control group.

## Etiology of Schizophrenia

Today, most researchers interpret the findings of a concordance of 0.25 to 0.5 in the many monozygote twin studies of schizophrenia as proof of a genetic trait and an environmental trait as well[13]. Obviously, the factor most dominant is the environmental, estimated from the size of the found concordances.

New information about the life as fetus comes from fetal regression, where adult patients and researchers engaged in regression back to the womb; a method practiced for many years by Tibetan Yogis[14], but until recently seen as impossible[15,16] as the fetus has no mature brain, and the early fetus no brain at all. The validity of such experiences has been tested and often the remembered facts were found to be actually correct[15,16], although this is still an issue of debate. The fetus being a conscious being interacting with the world, and actively adapting to the circumstances of life, is in severe contrast to the much more mechanical interpretation of embryonic development held by science until recently. If the subjective data about fetal adaptation is found to be valid, the twin studies must be reinterpreted. The twin studies are thus neglecting that monozygote twins are in very close contact to each other in the womb, and that the fetuses are actually aware, and communicating and sharing many subjective experiences in the womb, which might as well as bad genes explain the found concordance.

From the perspective of clinical holistic medicine (CHM), where patients go back into the womb very often, and where these experiences give meaning and value to the process of existential healing of the most early defenses, there is no doubt that environmental factors by far dominate the genetic influence. This is the reason why schizophrenia and other mental illnesses seemingly founded already in the womb in many cases can be healed in holistic existential therapy.

Under all circumstances, it is important to stress that the often-heard argument that schizophrenia is in fact an incurable genetic disease documented by the many monozygote twin studies is not valid; the concordance is much too low to substantiate this view. Data are quite opposite in favor of the view that schizophrenia primarily is a psychosocially induced disturbance, which therefore possibly can be cured by the persisting psychosocial problems from the patient's past being solved. Future clinical research will tell us if our optimistic interpretation of data is substantiated.

## Diagnostic Guidelines (ICD-10, F20 Schizophrenia)

In the ICD-10 system, the normal requirement for a diagnosis of schizophrenia is that a minimum of one very clear symptom (and usually two or more if less clear cut) belonging to any one of the groups listed as (a) to (d) below, or symptoms from at least two of the groups referred to as (e) to (h), should have been clearly present for most of the time during a period of 1 month or more. Conditions meeting such symptomatic requirements, but of duration less than 1 month (whether treated or not) should be diagnosed in the first instance as acute schizophrenia-like psychotic disorder and are classified as schizophrenia if the symptoms persist for longer periods.

a. Thought echo, thought insertion or withdrawal, and thought broadcasting;
b. Delusions of control, influence, or passivity, clearly referred to body or limb movements or specific thoughts, actions, or sensations; delusional perception;
c. Hallucinatory voices giving a running commentary on the patient's behavior, or discussing the patient among themselves, or other types of hallucinatory voices coming from some part of the body;
d. Persistent delusions of other kinds that are culturally inappropriate and completely impossible, such as religious or political identity, or superhuman powers and abilities (e.g., being able to control the weather, or being in communication with aliens from another world);
e. Persistent hallucinations in any modality, when accompanied either by fleeting or half-formed delusions without clear affective content, or by persistent overvalued ideas, or when occurring every day for weeks or months on end;
f. Breaks or interpolations in the train of thought, resulting in incoherence or irrelevant speech, or neologisms;
g. Catatonic behavior, such as excitement, posturing, or waxy flexibility, negativism, mutism, and stupor;
h. "Negative" symptoms, such as marked apathy, paucity of speech, and blunting or incongruity of emotional responses, usually resulting in social withdrawal and lowering of social performance; it must be clear that these are not due to depression or to neuroleptic medication;
i. A significant and consistent change in the overall quality of some aspects of personal behavior, manifested as loss of interest, aimlessness, idleness, a self-absorbed attitude, and social withdrawal.

## Diagnostic Criteria (DSM-IV Code 295 Schizophrenia)

The DSM-IV system basically focuses on the same symptoms, but uses a slightly different system:

A. Characteristic symptoms*:* Two (or more) of the following, each present for a significant portion of time during a 1-month period (or less if successfully treated):

1. Delusions
   2. Hallucinations
   3. Disorganized speech (e.g., frequent derailment or incoherence)
   4. Grossly disorganized or catatonic behavior
   5. Negative symptoms, i.e., affective flattening, alogia, or avolition

*Note*: Only one Criterion A symptom is required if delusions are bizarre or hallucinations consist of a voice keeping up a running commentary on the person's behavior or thoughts, or two or more voices conversing with each other.

B. Social/occupational dysfunction: For a significant portion of the time since the onset of the disturbance, one or more major areas of functioning, such as work, interpersonal relations, or self-care, are markedly below the level achieved prior to the onset (or when the onset is in childhood or adolescence, failure to achieve expected level of interpersonal, academic, or occupational achievement).
C. Duration: Continuous signs of the disturbance persist for at least 6 months. This 6-month period must include at least 1 month of symptoms (or less if successfully treated) that meet Criterion A (i.e., active-phase symptoms) and may include periods of prodromal or residual symptoms. During these prodromal or residual periods, the signs of the disturbance may be manifested by only negative symptoms or two or more symptoms listed in Criterion A present in an attenuated form (e.g., odd beliefs, unusual perceptual experiences).
D. Schizoaffective and mood disorder exclusion: Schizoaffective disorder and mood disorder with psychotic features have been ruled out because either (1) no major depressive, manic, or mixed episodes have occurred concurrently with the active-phase symptoms; or (2) if mood episodes have occurred during active-phase symptoms, their total duration has been brief relative to the duration of the active and residual periods.
E. Substance/general medical condition exclusion: The disturbance is not due to the direct physiological effects of a substance (e.g., a drug of abuse, a medication) or a general medical condition.
F. Relationship to a pervasive developmental disorder: If there is a history of autistic disorder or another pervasive developmental disorder, the additional diagnosis of schizophrenia is made only if prominent delusions or hallucinations are also present for at least a month (or less if successfully treated).

As both diagnostic systems use almost the same patognomonic criteria, it has been quite easy to establish the diagnosis with great certainty for almost a century.

## CHM and Schizophrenia

In CHM, schizophrenia is seen as the most extreme state of lack of sense of coherence[17–25]. The purpose of CHM is to rehabilitate the sense of coherence through healing somatoform and psychoform dissociation, and rehabilitation of the patient’s physical, emotional, mental, and spiritual contact with other people through the channels of body and mind. To obtain this polyvalent effect, the standard short-term psychodynamic psychotherapy (STPP), which traditionally focuses on the patient’s emotional mind and sexuality, is complemented with bodywork (of Marion Rosen type[26]) and philosophy of life[27–36].

CHM uses the life mission theory[25,37–42] to understand the degeneration of the self into the ego, the shadow, the evil side of man, the loss of libido and talents through the repression of painful emotions, and the loss of existential coherence through the loss of energy, meaning, light, and joy[25]. CHM uses an expanded toolbox[43] and four central healing principles[44–46]:

1. Induce healing of the whole existence of the patient (also called salutogenesis)[17,18,45,46], not only his/her body or mind. The healing often includes goals, such as recovering purpose and meaning of life by improving existential coherence and ability to love, understand, and function sexually.
2. Adding as many resources to the patient as possible[6,7,15–18,45,46], since the primary reason for originally repressing the emotionally charged material was a lack of resources — love, understanding, empathy, respect, care, acceptance, and acknowledgment, to mention a few of the many needs of the little child. The principle was also to use minimal intervention as necessary, first using conversational therapy, then adding philosophical exercises if needed, then adding bodywork or, if needed, adding role plays, group therapy, and finally when necessary in a few cases, referring to a psychiatrist for psychotropic intervention. If the patient was in somatic or psychiatric treatment already at the beginning of the therapy, this treatment was continued with support from the holistic therapist.

3. Using the similarity principle that seems to be a fundamental principle for all holistic healing[45,46]. The similarity principle is based on the belief that what made the person sick originally will make them well again, when given in the right, therapeutic dose. This principle often leads to dramatic events in the therapy, and to efficient and fast healing, but seems to send the patient into a number of crises that must be handled professionally[47–49]. The scientific background for a radical and fast healing using the similarity principle is analyzed in several studies[50–55].
4. Using Hering's Law of Cure[50–55], supporting the patient going once again through all the disturbances and diseases, in reverse order, that brought the patient to where he or she is now. Other important axioms of Hering's Law of Cure are that the disease goes from more to less important organs, goes from the inside out, and goes from upside down. The scientific rationale for the last three axioms is less clear than for the first: the patient must go back to the beginning of his time line to integrate all the states and experiences s/he has met on her/his way to disease. Going back in time is normally done through spontaneous regression in holistic existential therapy.

By using these tools and principles, many physical, mental, sexual, and existential problems can be addressed[1–10,46–48,57–81] with satisfactory results[82–86]. Even severe mental problems can be alleviated[4,5,85] and, in several cases, patients with schizophrenia have been cured (see cases below)[5,85,87,88], but we still need research to document the effect of CHM with a large group of well-diagnosed, schizophrenic patients. Since the patients we have been working with might be in the more healthy part of the schizophrenic spectrum (see the cases below), we have been warned that many schizophrenic patients will be more difficult to cure.

## Clinical Holistic Treatment of Schizophrenia and Other Psychotic Diseases

While many of the psychiatric diseases can be treated efficiently with STPP, schizophrenia still seems to challenge the psychodynamic psychotherapist. The most difficult aspect of the therapy is how to engage the patient in the often quite unpleasant therapy. The autistic aspect of schizophrenia makes it difficult make contact at all; the severe emotional disturbances makes it difficult develop a normal relationship with the patient; and the severe thought disturbances makes it difficult to talk in a coherent and meaningful way and to make a therapeutic contract with the patient. The patient's hallucinations make it difficult to share a common reality; delusions gives birth to strange behavior and idiosyncratic logic; apathy, paucity of speech, and blunting or incongruity of emotional responses results in general social withdrawal and lowering of social performance, often leaving the patient without any resources and thus taking the patient completely out of the "game of life".

So where to start? The goal is to help the patient back into the world as a contributing person; the practical solution of CHM is using a "the triple handle": the patient is contacted through body, mind, and total being (spirit) at the same time. The therapy uses whatever aspect the patient offers for getting into contact and achieving a proper therapeutic response. The fundamental goal of existential therapy is always to induce salutogenesis, or existential healing. Existential healing happens when the patient intends to heal and gets the resources needed for the healing process from the outside at the same time. During the process of healing, the patient will heal in three dimensions:

1. The outer world — healing relations and ability to function
2. The inner world — healing emotions, philosophy of life, and existential problems
3. The presence — healing the psychoform and somatoform dissociation through contact, communication, and development of the vital sense of coherence

The therapy must have a balanced focus on these three dimensions at all times.

## A Standard Course of CHM

With severely mentally ill patients, existential healing happens according to the metaphor of metamorphosis[78]: The patient is like a caterpillar, getting into the pupae stage to remember her true nature and transform, and finally reappearing like the butterfly. The phases of the existential healing seen through this metaphor are depicted in Fig. 1.

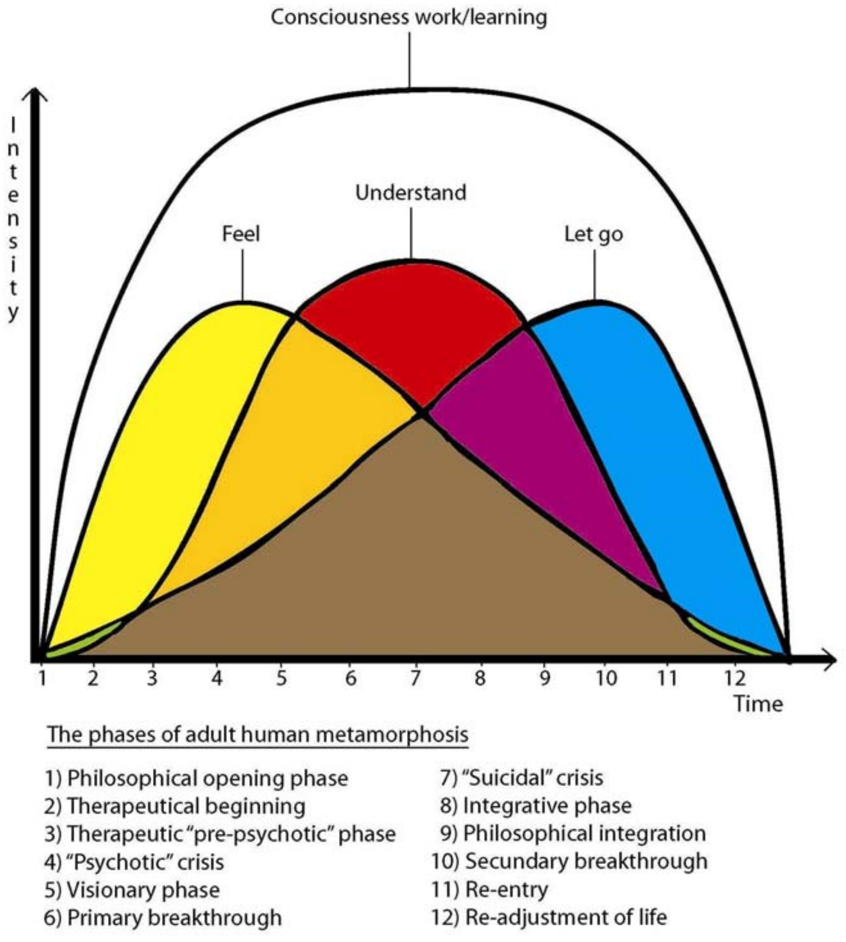

**FIGURE 1**. The therapeutic arc of transformation. Please notice that there are three existential crises connected to the process of healing from schizophrenia: (1) loss of old, safe self, and survival mechanism (giving up the splitting), often (re)experienced by the patient as a psychic death (number 4) in the figure; (2) the visionary phase, often called the healing crisis and often experienced as "holy madness" by the patient (number 5 and 6); and (3) the most intense, existential crisis of "to be or not to be", often experienced as a suicidal crisis by the patient (number 7), where the basic value of life is considered, and where the patient finally must choose life as it is[78].

Please notice that the patient supported by holistic therapy is not getting psychotic, mad, or suicidal in the crises mentioned in the legend of Fig. 1. The names refer to the subjective experience of the phases of healing, not to the patient's external and objective behavior. Actually, the patient is normally coming back to life during these crises, but the emotional intensity demands the most intense support and holding, often by several therapists at a time. The use of the concepts in CHM is thus very different from the use of the same concepts in standard psychiatry, as the concepts refer to the subjective experience, not to the objective symptoms.

When it comes to the most ill schizophrenic patients, it might be impossible to give the holding and support needed at all times, so it might be that we actually see psychotic relapses during therapy with this group of patients; such relapses might also be understood as a habitual pattern of defensive behavior, and as a re-experiencing and reintegrating of earlier psychotic events.

## Key Concepts of CHM in Relation to the Treatment of Schizophrenia and Other Psychotic Illnesses

A. Personal development of quality of life and insight in self — To engage a severely mentally ill patient in therapy, the fundamental metaphor must be easily understood and appealing. The concept of personal development is doing the job for most patients. The rehabilitation of the patient's character has been alpha and omega since Hippocrates[1].
B. Body-mind-spirit-existence (wholeness) — Holistic therapy makes it possible for the therapist to work with the whole patient, not only his/her mind. To define the therapy as holistic will open up for the therapist's use of bodywork, philosophical exercises, etc. that immediately mobilizes patient resources that cannot be activated within the standard frame of psychodynamic psychotherapy.
C. Purpose of life – good and evil[41] — To engage the patient in a deep self-exploration looking for the hidden purpose of life is often very helpful with the mentally ill patient. To explore the shadow (the good purpose reversing into the evil intent often troubling the patient) might be the door to a common understanding of the world, the illness, and the therapy – the latter as the way out of the underworld, in which the patent finds him/herself caught.
D. CHM: holding and processing — Love and support is necessary for the patient to relax, lean in, and assimilate the offered resources to get the process of healing going. The image of the therapy as the therapist keeping a firm grip of the patient in one hand, but beating him with the other (i.e., the good and the evil parent in one) often makes much sense to the patient.
E. Spontaneous regression, symbolic processing, death-rebirth, fetal regression, re-experiencing conception — When the patient receives the resources needed for existential healing, he/she will go back to the painful moments of childhood where he/she repressed large parts of consciousness to survive (the trauma causing the patient's splitting). We call this process spontaneous regression. It can take the patient all the way back to the first years of life, to birth, even into the womb and all the way back to conception. Even if this gives no sense for a skeptical mind, this is what the patients often experience in therapy, as first noticed by Grof[15,16], and before that by the tradition of Tibetan Buddhism[14] and other ancient mystery schools. The re-experience of conception often gives a strong feeling of direction in life and of primary talent (the life mission[25,37–42]).
F. Rehabilitation of patient's talents of sex, consciousness, and love — The primary talent of the patient is related to body, mind, and spirit, and thus to sexuality, consciousness, and love[39]. Many researchers in the treatment of schizophrenia have focused on rehabilitation of the patient's sexuality; Wilhelm Reich, Harold F. Searles, and many other outstanding therapists believed that the main etiology to schizophrenia and most other mental diseases was the patient's unconscious repression of genital sexuality. We believe that the ability to love and understand is as important for mental health as the ability to function sexually.

G. From isolation and meaninglessness to contribution and sense of coherence — One extremely important issue in CHM is the rehabilitation of the sense of coherence. This is achieved by rehabilitation of the patient's constructive attitude and behavior towards other people and the surrounding world. Using the talent for being of value to others is the essence of love, and when the patient is able to love and contribute, he/she is often well again.

H. Philosophy of life – from negative, destructive, and delusional to rational and constructive understanding — Rehabilitating the patient's positive and constructive, natural philosophy of life and rational thoughts is the most important condition for a permanent cure[5]. The negative philosophy is actually a sum of hundreds of negative decisions through life, each holding on to a negative emotion from a painful life event. In the therapy, as the emotional charge is removed from the personal history, a constructive and positive philosophy of life slowly reappears.

I. The metaphor of metamorphosis – psychic death, transformation, and re-entry into the world — The schizophrenic patient's flow of consciousness is most often filled with negative issues, such as death, illness, rotten flesh, urine and feces, perverted sex, incest, homosexuality, rape, sadomasochism, pedophilia, violence, all kinds of evilness and torture, darkness, and death. The acceptance of all these elements and the rational explanation of these elements' dominating position in the patient's mind is often experienced as a great relief. The therapist who is familiar with all these emotionally difficult subjects and accepts them allows the patient to share for the first time in his or her life the true content of the stream of consciousness. These elements have been classical elements of the traditions of Tantra[14,89,90] and other mystery schools, but most people do not accept them, even when they are present in their experiential life, as they are a strong taboo of contemporary Western culture and society. To allow the patient to engage fully in the exploration of the shadow side of modern man and Western culture is often a major key to healing.

J. The healing crisis, hole madness, and crazy wisdom — With hundreds of thousands of hits in a yahoo.com or google.com search, these concepts are well integrated in contemporary spiritual culture. Originally coming from the orient, most people who engage in serious, spiritual development now know them. They signify the wisdom coming from within and materializing through dreams and visions in states of expanded consciousness. These states are introduced by meditation, holotropic breath work, fetal regression, or other intense self-explorative techniques. Most unfortunately, many psychiatrists do not know them, and do not recognize when the patient has entered a healing crisis that is an obligatory part of any recovery from schizophrenia. Instead of supporting the patient in the vital project of self-exploration, these doctors take the patient "down" from the expanded state of consciousness with antipsychotic drugs, thus arresting the spontaneous healing taking place.

K. Wounded healer — The only way a therapist can truly help his patient is by letting him be the tool for the patient's healing. To do that, he must admit that he is only a human being himself; he must admit that he has all human failures and faults himself; he must admit that he is also a wounded child, and not yet perfect; he is also in the process of human development and in that sense, very much in the same position as the patient. Only by sharing the common human conditions, and being together in a true fellowship, can the therapist give sufficient support and holding for the patient to heal the core of his existence.

L. Confronting the evil in whatever form — We are as evil as we are good. We pretend that we are good, or we repress our evil side to our shadow, but when we are true and honest, we must admit that only by choosing the good and abandoning the evil can we become decent and ethical human beings. Owning the evil is mandatory for the schizophrenic patient, as splitting of the evil side of man is the most common kind of splitting. Secondary is the splitting of the body and sexuality, or of the divine, spiritual aspect of the patient's being.

M. Negative decisions and axiomatic thinking — A trauma always consists of an unbearable feeling and a life-denying, negative decision. This decision is a generalized justification that works as an axiomatic basis for the interpretation of the patient's world. These negative decisions are behind the delusions that the patient presents, and only by taking the patient all the way back to the fundamental emotional pain of the trauma fixating the negative belief, can the patient get rid of his delusions. Table 1[78] is a list of the most important negative and life-denying sentences that were released during a therapy session, when the patient healed her borderline condition. The sentences were the essence of the gestalts that were integrated into the therapy; they are both feelings and thoughts at the same time, making them extremely to the point of the experience. (*Note*: While letting go of these decisions, she healed her existence and recovered from a severely dysfunctional state. The traumas resulted from sexual abuse in her childhood[78]).

**TABLE 1**

| | |
|---|---|
| 1. | Nobody likes me |
| 2. | I don't want to know it |
| 3. | I can't stay anywhere |
| 4. | I am out in the space |
| 5. | This is unreal |
| 6. | I am empty |
| 7. | I am hollow |
| 8. | It is not me |
| 9. | I can do nothing |
| 10. | I do not need you |
| 11. | I need nobody |
| 12. | I cannot do that |
| 13. | I am a failure |
| 14. | There is no room for me |
| 15. | I am nothing |
| 16. | It is absurd |
| 17. | She is a schizophrenic |
| 18. | I am a schizophrenic |
| 19. | I do not deserve to live |
| 20. | Why didn't they kill me? |
| 21. | I get smashed up |
| 22. | I go to pieces |
| 23. | She is going to kill me |
| 24. | He is going to kill me |
| 25. | They are going to kill me |

N. The fundamental conditions of existence (the "philosophical existentials") — Every person is alive and therefore going to die, which creates anxiety; every person is autonomous and therefore basically alone; and every person intends to contribute in love and therefore fails and suffers. These three existential pains are inevitable and every conscious person on Earth must bear these fundamental conditions to be a human being. This must be thoroughly understood by the patient, who normally thinks that these are unique and horrible conditions that God gave his/her poor soul.

O. Precautions — To make psychodynamic therapy with CHM a success, there are certain issues of crucial importance:

1. Avoid higher ages — The older the patient, the more difficult the therapy.
2. Avoid institutionalization — The longer the time spent at an institution, the more difficult the therapy.
3. Avoid pharmaceutical drugs — The higher the dose and the longer time treated, the more difficult the therapy. If the patient is already a psychiatric patient on antipsychotic drugs, but wishes a CHM treatment, this situation must be handled carefully. For the CHM therapy to be efficient, patients need their full intellectual capacity; as they often experience the antipsychotic drugs as sedating, they will often insist on coming off the antipsychotic drugs. We find this reasonable and suggest that it happen gradually, with the patients well supported, in a number of well-controlled steps. Sometimes the patients want to get off the drugs right away, which might make them re-enter their original psychosis; this can be very therapeutic, but only if there are sufficient therapeutic resources available (several therapists and holders 24/7). Most often in our experience, the patients who wants to get off the drugs do not have compliance and only pretend to take the drugs, making this problem much less than it might seem.

4. Avoid giving a psychiatric diagnosis if possible — The more the patient thinks of him/herself as “schizophrenic”, the more difficult the therapy. If they already have a diagnosis, this must be carefully explained as something temporary and not something final.
5. Avoid negative, philosophical implants — The more hopeless the patient believes his/her situation to be, the more difficult the therapy.
6. Handle the transference of love and sexuality with great care — The more the patient feels rejected and judged, the more difficult the therapy.
7. Handle the acute psychosis with patience and love — The more severe the trauma from losing control to psychosis is, the more difficult is future therapy.
8. Prevent suicide attempts — The more attempts, the more difficult the therapy.
9. Prevent self-mutilation, self-abuse, humiliation, and loss of: friends and social network, social reality (e.g., school), family, self-worth, and self-respect — The deeper the patient falls, the less resources and the more difficult the therapy. One efficient way to do this is by having a person (a “mentor” or a “sponsor”) enter into a close, intimate, supportive relationship with the patient, for a while being his or her “best friend”.

## CASE STORIES

### CASE 1: Psychotic illness in a 7-year-old boy caused by implanted philosophy and cured in 1 h[5]

A 7-year-old boy was taken into therapy because he washed his face and hands every 30 min, day or night, obsessively. He became furious if somebody stopped him. VandenBos[5] discovered, during a 1-h session that cured the boy, that a doctor had implanted the understanding that the nerves were worms living under the skin. Just clearing the misunderstanding cured the boy. If VandenBos had not examined the boy and cleared this up, he would have been taken to a psychiatric ward, and the normal procedure and the trauma of the hospitalization in itself could easily had given symptoms that would have confirmed the tentative diagnoses of schizophrenia. This could easily have turned the child into a chronic patient as VandenBos concluded[5]. If only all cases were this easy!

### CASE 2: Schizophrenic 30-year-old male cured by 3 years of CHM

A 30-year-old man lived a protected life on a small island. From early childhood, he lived with his grandparents, as his own parents could not raise him. When he came to the clinic, he had suffered for years from emotional blunting and an almost complete withdrawal from other people (autism); he had severe delusions of living in outer space, combined with strongly peculiar obsessive, catatonic behavior (standing fixated on top of telephone boxes to be close to other people), combined with the symptoms of subjective depersonalization and derealization. At the time he started therapy, he considered committing suicide, but was also very scared of dying (ambivalence). He was severely visually hallucinated. He was thinking according to his own personal logic. He sometimes used prostitutes, which he mostly paid to talk with him, which was his only contact with the other sex. On the quality of life scale (QOL5) he rated himself as “bad” (4 on a scale of 1–5, 5 being the bottom of the scale); he rated his self-evaluated mental health as “poor” (4 on the 5-point Likert scale).

This patient who had persistent hallucinations (e), catatonic behavior (g), negative symptoms (h), significant and consistent change in personal behavior, and social withdrawal (i), was diagnosed /F20 Schizophrenia/ according to ICD-10. He was presumably catatonic schizophrenic, but this diagnosis was not used.

At the beginning of the CHM therapy, he was only treated philosophically, as philosophical discussions were the only contact possible with him; he was given books to read and then the content discussed with the therapist (SV). The books were about philosophy of life, such as those by Buber[91] and Chopra[92]. The patient was clearly intelligent (IQ not measured) and this resource was used. In the next psychodynamic phase of the therapy, he came into contact with his feelings and went through the existential crisis that is standard with CHM (see Fig. 1), and was now given the challenging exercise to confront his enormous anxiety. He had to visualize that the anxiety was a fire in which he was burned and purified. We did this successfully. Then he was supported in confronting his visual hallucinations, which he also managed to do.

The next theme was to support him in confronting his problems with women and sexuality. He let go of his use of prostitutes and used his sexual drive to go to town and meet and talk to women; after months of demanding, behavioral training, he finally managed to behave socially acceptable on a dance floor, and finally got a girlfriend and a semi-normal sexual life.

After 4 months of therapy, 1 h/week, and intensive writings of dozens of full pages in the form of a patient’s diary, the patient was able to use the concepts from therapy and from the books he had been reading in his thinking; and for the first time in his life, he had a valid language for his strange, alienated perceptions. This made him reflect upon himself and he realized how sick he really was: “Today I was surprised to realize that I really have hallucinations…I jumped into a state of death, with no ability at all to express my sense of I-Thou”.

The middle phase of therapy consisted of holistic bodywork (a female holistic physiotherapist) to help him; she managed to take him back to the most painful part of early childhood, when he was abandoned by his mother (and father), and in healing these old, deep wounds on his soul, he finally came back into normal, emotional contact (induction of salutogenesis through the parallel healing of psychoform and somatoform dissociation).

The third and final part of the therapy focused on supporting the patient in creating a new philosophy of life that allowed him to step into character and use his bodily, mental, and spiritual talents[25,42].

The CHM case record for this patient is close to 100 pages, most of them written by the patient. They demonstrate the complete transformation in his logic, value system, relationship with himself and the world, and sexuality. He is not yet able to love other people in a normal way, but he is still recovering, and there is no doubt that his sense of coherence is being rehabilitated. At the end of therapy 3 years after he started, he rated his QOL5 as 3 (intermediate) and his self-evaluated mental health as “good” (2 on the 5-point Likert scale). He had hallucinations no longer (symptom e), his catatonic behavior was gone (g), his negative symptoms were gone (h), his significant and consistent change in personal behavior and social withdrawal (i) was radically improved. Thus, he no longer fit the diagnosis of ICD10-F20 Schizophrenia (or DSM-IV Code 295). The patient was cured after about 40 sessions (one every third week) of CHM therapy during 3 years. He continued in therapy for another year to improve his general ability and after that, he went to university to take his MSc in computer science, where he did well.

## CASE 3: Schizophrenic 15-year-old male cured by 2 years of CHM

This 15-year-old boy was brought to the Research Clinic for Holistic Medicine by his parents, who were not satisfied with the results from several years of psychiatric treatment with neuroleptic drugs and hospitalization at an institution for violent young mentally ill patients. The parents believed that the drugs did not help him and that he got too much medicine, which sedated him without curing him, and they did not see any improvement as time went by.

He had been extremely violent in school, clearly psychotic, and living with strong delusions of being another person, a German soldier from the Second World War, whose life he clearly remembered. The things he told from his personal story were strongly worrying; among other things, he had been just about to kill a friend at school, of which he was rather proud as it proved that he was the strong Nazi soldier he believed himself to be. He hallucinated and saw his father as an English pilot he remembered that he killed during the war.

We accepted him into CHM therapy and the municipality accepted to pay for his therapy (yearly cost for the institution was about 150,000 Euro, the cost of treatment in our clinic about 4,000 Euro a year). According to ICD-10, he was clearly having the following symptoms:

d. Persistent delusions that are culturally inappropriate
e. Persistent visual hallucinations occurring every day for months on end
h. “Negative” symptoms, such as blunting and incongruity of emotional responses
i. A significant and consistent change in the overall quality of personal behavior

According to ICD-10, his diagnosis was thus /F20 Schizophrenia/. In the beginning of the therapy, the patient was very vulnerable and weak in emotional contact; he went through a big transformation process and astonishingly ended up on his feet as a healthy young man who is doing well socially and at school. He stopped the medicine during the first months of therapy by himself, without sharing this with anybody, and obviously without needing it any more.

In the therapy, evilness was the big theme and after months of confrontation of his shadow (his evil side), he ended up by choosing to be good and let go of his idea of being the German warrior. After this fundamental shift in his personal philosophy, he was able to regain empathy and emotional contact slowly; he went back to school, where he is doing really fine and caching up on the 3 years he missed. After a few episodes of moderate violence (fighting with the other boys), he managed to get his anger and fury under control, and continues to develop into a well-functioning boy, i.e., fitting in and being appreciated by the other kids. He got a girlfriend, which is very good for his self-esteem and feeling of being a young man, growing up and participating in society. He is now of great help to his family and his progress is highly appreciated.

In holistic therapy, using also bodywork, the patient’s psychoform and somatoform dissociations were healed and his sense of coherence slowly recovered. After 2 years and about 40 sessions, he was out of regular therapy, but continued for follow-up sessions every 6 months. About a year after the therapy ended, he received the following statement from his teacher at school:

> “PATIENT NAME has in relation to diligence and cooperation been doing satisfactory work. He has been positive and very engaged in the subjects. In the class room PATIENT NAME has contributed creating a good and positive atmosphere as a good listener and friend. PATIENT NAME has furthermore good relations to the school staff. The school therefore gives him the best recommendations.”

His delusions, hallucinations, incongruous emotional responses, and behavioral disturbances (including his tendency to violence) have now completely gone. Thus, he no longer fits with the diagnosis of ICD10-F20 Schizophrenia (or DSM-IV Code 295).

## Challenges for the Therapist

Working with schizophrenic patients gives the therapist all the challenges you can wish for… and a little more. Only if you are up to an intense project of accelerated self-development and an exploration into your own shadow for deep self-insight will you find it really rewarding to work with this group of patients. Below is a list of the challenges that we have met in our work with these patients.

- All symptoms and symbols are meaningful — The therapist must contain his confusion because, in the end, all questions will be answered, but in the most surprising ways.
- The sexual content of the patient's unconscious will be explicit — Most traumatic events shared by the patient refer to real events, whether incest or rape[5], and you must be worth your salt as a therapist when the patient brings this to the sessions.
- Implanted philosophy — The most important gift the therapist can give to the patient is his optimism about the patient's ability to heal and recover[5]; often you can hardly believe that the patient has a chance, but you must choose to believe it.
- Never expect your patient to be grateful or to acknowledge improvements — When a problem is gone, it is out of the patient's mind forever, so keep a thorough case report and do not expect patients to remember their progress or even thank you for curing them when they are cured[5].
- The more healthy the patient gets in the process, the more troubled will the patient's feelings and emotions be — An example from Karon and VandenBos[5] is a girl that begins therapy completely unable to have sex and after successful therapy, starts complaining about not liking her new boyfriend much and not receiving much pleasure from sex. She is not grateful at all for being able to have a boyfriend now, but is angry over things that are not right and perfect yet. This is clearly a patient healing. Only by keeping a good case record can this progress be appreciated by the therapist.
- If the patient is being more emotionally labile, angry, expressive, and difficult, it is a good sign of healing — Most therapists, as most parents, do not really appreciate people getting more powerful, selfish, and demanding, but it is a fine sign of individuation. Emotional crises will get more and more intense, and more and more frequent. That is the patient coming back to life, which is always painful. Often, the awakening sexuality will give the patient strong sexual feelings and a most uncomfortable and embarrassing behavior, especially when the patient is transferring sexuality on the therapist. This is not the patient getting more sick, but the patient getting into a developmental state similar to retarded puberty, and the sexuality of the patient must not be condemned or repressed if the therapist wants the patient to recover.
- The patient's borders are often transgressed and badly defined — Most patients have never had a private room in their life, and the establishment of such a sacred, personal space is a necessity for the patient to recover his/her identity. To support the patient in having such a space, the therapist must accept that the patient has secrets.
- Counter transferences are often difficult — The schizophrenic patient is often living in a self-made hell that we do not want to look into; when we do so, our empathy causes us unbearable pain. Sharing the position of feeling reduced from being a person to being something like a mistreated animal, or even a thing without life and existence, especially puts us in severe agony. The patient's defenses will make the patient do everything to hinder that we get close to the patient, so very often the patient will make him/herself dirty, ugly, repelling, etc., just to make us keep an emotional distance. Some patients use urine, feces, saliva, dirty clothes, etc. to make themselves less appealing. Psychotic patients present their emotional, sexual, etc. material in the most raw and least socially acceptable form for the same reason: To prove to themselves that we do not like them at all – the same way they felt rejected and not loved by their parents.
- Risking murder and suicide — Psychotic patients do not have the inhibitions that normal people have. Sometimes they kill themselves and sometimes they kill others. The psychology of a murder is often a patient that cannot accept angry impulses (cannot contain conscious anger) and represses these impulses until the day he explodes[5]. Often, sexuality and jealousy are central issues here. Most schizophrenic patients are not dangerous at all, but the therapist should use his intuition and common sense, and be very careful when the patient feels like a “bomb” of repressed anger and is living in a conflict involving jealousy.

- Most patients, whether psychotic or borderline, consider committing suicide during the therapy[5] — Actually, the suicidal crisis must be seen as an important part of the therapy because this is when the patient really chooses life for the first time. Fortunately, the consideration hardly ever leads to the action of committing suicide, but the danger of the patient committing suicide must be considered in every consultation, and if suicidal thoughts are present, the issue must be addressed directly, to support the patient in learning from the crisis and preventing suicide.
- Suicide, when actually committed, often materializes the desperate intent of revenge — The calculation is that if the patient kills him/herself, the person(s) who failed the patient will be really sorry! Most often, suicidal attempts are nothing but a cry for help – the young girl takes four sleeping pills and knows she will be found with the suicide letter in her hand. Much more seriously endangered is the 60-year-old male and recently retired patient that demonstrates the “presuicidal syndrome”[93].
- It is very important to notice that research has shown that ECT (electroconvulsive therapy) does not prevent suicide, but just postpones it[94] — The prescription of antipsychotic drugs is very dangerous in the case of a suicide threat, as everybody knows that drugs are often used to commit suicide; drugs also take the patient into depression, enhancing the emotional drive against suicide[94].
- Risking violence from the patient — Therapists are often exposed to threats and, even on rare occasions, to violence when working with schizophrenic patients. Karon and VandenBos[5] described a patient putting a pin into her knee during a session, with the intent of making the therapist hate her. For most schizophrenic patients, love and kindness is hard to stand; hatred and anger are better, and emotionally cold rejection and even total lack of interest makes the patient feel safe.
- Mastering controlled fail of the patient — The reason is that most schizophrenic patients come from severely dysfunctional families, where they have survived without getting the love and close contact that is a normal part of other children’s upbringing. The ability of the therapist to fail the patient intentionally, using the principle of similarity, is a central condition for being able to cure. Most therapists engage themselves in their patients and when they start to know them, they often also come to like them. In this case, holding is appropriate and processing must follow. Here the therapist must shift between being the good father/mother of the patient and the evil father/mother. Many therapists find this shifting necessary for supporting the schizophrenic patient most difficult.
- Containing self-mutilation — Often, schizophrenic patients are self-destructive and this must be contained by the therapist. When this is directly addressed, the reason for the impulses is often understood and self-mutilation stops. Karon and VendenBos described a self-mutilating patient that cut his arm using broken glass from a glass bulb[5]. Karon used the occasion to give the patient credit for stopping before the artery was cut: “I am proud of you. You stopped cutting yourself before somebody else had to stop you”[5]. This direct confrontation without any judgment made the patient stop his self-mutilation.
- Patient that will not eat — Karon and VandenBos[5] recommended psychotherapy during a meal as the key to solving this problem.
- Sleeping disorders — Karon and VandenBos[5] recommended full acceptance of the state as a solution and recommended that the patient not sleep, but just to lay down for 8 h of resting, without getting up during the night. Most patients argue that this is very boring, and the answer is to acknowledge the boredom and reassure the patient that he/she will be able to function the next day.
- Illegal drugs — Most schizophrenics are not attracted to heroin or other similar drugs, but if they are hallucinating, they can be very interested in hallucinogenic drugs that can enhance the hallucinations and give them insight into the nature and hidden courses of the hallucinations; regular use of high doses of hallucinogens are to be considered self-destructive[5] and the therapist should warn the patient against the negative consequences of abuse, like bad trips (drug-traumas) and flash backs[5].

## DISCUSSION

Schizophrenia is often regarded today as the most difficult disease to treat with psychotherapy, especially when the patient is in a psychotic state or hospitalized[95]. Normally, therefore, the schizophrenic patient is treated with a drug and only when the patient is back in a “normal” and communicative state of being can additional psychotherapy be introduced.

Unfortunately, only one in four or less reacts well to, for example, chlorpromazine, and one in two gets serious physical side effects[12]. The drug is also suspected to provoke severe mental adverse effects with dysphoria, depression, and even the feeling of “being like a zombie”[96]. In addition to that, a spontaneous overmortality of up to 150% has recently been documented by the Danish Health Authorities for specific subgroups[97].

The standard psychiatric treatment is known not to cure schizophrenia, but only to reduce some of its symptoms, and schizophrenia is considered by most psychiatrists to be a chronic and incurable disease. The standard psychiatric treatment is thus a rather imperfect treatment for schizophrenia and alternatives must be sought. Luckily, the last decade has given us documentation that psychodynamic psychotherapy is efficient with mental diseases[8–10] and presumably also with schizophrenia[5,85,87,88].

Psychotherapy in patients with schizophrenia has been considered more difficult than the treatment of any other group of mental illnesses, due to the triad of autism, emotional “flattening”, and uncorrectable delusions. When the contact with the patient is very poorly anchored in mind, feeling, and body, as we see it in patients with severe somatoform and psychoform dissociation, the possibility to have an impact on a patient is rather limited. If the patient is psychotic and not oriented in time, space, and own data, this becomes even harder. Seen from the therapist’s perspective, there is no “handle” to get hold of the patient and start turning him/her back towards reality.

To solve this problem, we have expanded STPP with bodywork and philosophy of life exercises[47-49], giving the therapist a possibility to get in contact with the patient at a physical, mental, and spiritual level at the same time. The triple grip on the patient allows us to work with even the most psychotic patient, although therapeutic progress is not always visible in the beginning of the therapy. We have observed in the patients that come to our clinic that three things are associated with a good outcome of therapy: the younger the patient is, the shorter time a patient has spent in a psychiatric ward; the shorter time the patient has been on antipsychotic drugs, and the shorter time the patient has been ill, the easier it is to help. We have worked with 10 insufficiently diagnosed schizophrenic/schizotypical patients and about 30 borderline patients[44], and have noticed a certain path of healing for these patients that often takes a quite dramatic form when treated with CHM, with a quite number of severe existential crises before healing existentially (see Fig. 1).

We have been working with schizophrenic patients directly who have the deluded thoughts, the flattened emotions, and the autistic withdrawal from the world. We have found that all these symptoms that characterize the schizophrenic patients can, in most cases, quite easily be traced back to severely dysfunctional family patterns of the patient’s childhood. The method of spontaneous regression, taking the patient into fetal regression, has especially made it possible to include the prenatal adaptation to the future family, which has given many of the missing pieces to a full psychodynamic understanding of schizophrenia. These adaptations also seem to be able to explain the concordance seen in the twin studies with schizophrenic patients, stressing the environmental factors and thus giving an alternative hypothesis to the hypothesis of genetic courses of schizophrenia.

The patient is often found in a severely dysfunctional state of being with very few, if any, stable, intimate, personal, confidential contacts. So helping the patient back to life is basically about inducing salutogenesis by rehabilitating a sense of coherence in two directions: inwards, towards life, and outwards, towards other people and the surrounding world. To induce the recovery of schizophrenia, the first thing to be done is to give the schizophrenic patient the optimal holding they need, to go back to confront old childhood traumas. In the therapy, we normally give them two “new” parents: a male and a female therapist who act as if they were the patient’s “good” and “bad” parents during therapy. One of the therapists is good, while the other is evil, taking the patient back by using the principle of similarity, while giving the patient all the loving and caring support needed for existential healing.

We have found that as soon as we are able to engage the patient in the therapeutic contract, the therapy starts to work. We have found that one in two of the schizophrenic patients (the limitation is our small sample) was cured during 1 year of holistic therapy. Unfortunately, the limited number is not sufficient for statistical analysis, and the diagnoses were not made strictly according to an international system like ICD10 or SDM-IV, so we are now conducting another experiment that includes a larger number of patients and more well-defined international diagnostic criteria.

Dissociation is a well-established phenomenon [DSM IV, 98–102] occurring in people who have experienced some form of trauma. Trauma can be defined as anything that overwhelms our resources[99]. According to the Diagnostic and Statistical Manual for Mental Disorders, Fourth Edition (DSM-IV; American Psychiatric Association, 1994), the essential feature of dissociation is a disruption of the normal integrative functions of consciousness, memory, identity, and perception of the environment. Much of the research on dissociation emerged from the identification of Post Traumatic Stress Disorder as a distinct condition. Van der Kolk et al.[98] identified core symptoms of PTSD as intrusions (thoughts, dreams, flashbacks), hyperarousal, and numbing. Dissociation is a real, biological phenomena arising from trauma, and both somatoform and psychoform dissociation must be healed to cure schizophrenia and other psychotic mental disorders.

It is still a matter of discussion if traumas are the underlying cause of schizophrenia; our success in healing a few schizophrenic patients by healing their traumas definitely points in this direction.

During the last two centuries thousands of therapists have succeeded in curing a fraction of their schizophrenic patients; we know of Philippe Pinel and his students, with their moral therapy cured patients, but as the diagnosis schizophrenia (or dementia praecox) was not available yet, we do not know the fraction of cured schizophrenic patients. When data on the treatment of schizophrenia became available, we see that 33% were cured and 33% radically improved, but the rest had only little improvement or were not helped by psychotherapy (Harold Searles statistics are documented in [103], the introduction). Similar results were produced by a number of remarkable therapists, such as Jung (1875–1961)[7], Adler (1870–1937)[104], Abraham (1877–1925)[105], Federn (1871–1950)[106], Harry Stack Sullivan and Frida Fromm Reichmann further developed by Will (1961)[107] and Searles (1965)[108], Schilder (1935)[109], Rosenfeld (1965)[110], Segal (1950)[111], Fairbairn (1954)[112], Guntrip (1969)[113], Perry (1961)[114], Lidz (1973)[115], Kernberg (1975,1976)[116,117], Volkan (1976)[118], Sechehaye (1951)[119], Rosen (1953)[120], Eissler (1952)[121], Arlow and Brenner (1964)[122], Giovacchini (1979)[123], Arieti (1974)[124], Bellak (1979)[125], Gendlin (1967)[126], Prouty (1976)[127], Gunderson and Mosher (1976)[128], and Karon and VandenBos (1981)[5]. But even before Pinel and the psychotherapists, the Hippocratic doctors healed the hysteric and mentally ill patients for millennia with holistic medicine.

In spite of this strong and scientifically well established tradition, many physicians today have come to believe that schizophrenia cannot be treated with psychotherapy, which has been discussed by Karon and VandenBos[5]. It seems that many physicians believe that it is very difficult and demands special skills, knowledge, talents, and training to make therapy with schizophrenic patients available. Karon and VandenBos argued that this is not the case, since everybody who cares about people and knows elementary psychotherapy can treat schizophrenic patients, and although these patients can be very difficult, some of them are easy to help and almost nobody is completely impossible to help[5].

## CONCLUSIONS

It is not new that psychodynamic psychotherapy and holistic medicine can help to cure schizophrenia and other psychotic mental disorders. In one study, Searles treated a group of 18 of the most heavy, chronically ill schizophrenics with a duration of illness of an average 9.2 years, and 2.3 years of hospitalization on average before entrance into the study. After therapy, 33.3% were cured ("remarkably improved" and out of hospital) and a further 38.9% were "remarkably improved", but still hospitalized[103]. Searles kept records of 600 patients for his research and continued to be optimistic throughout his life. It is generally believed that the earlier the treatment starts, the better will be the therapeutic results. Based on the available data, we estimate that schizophrenic patients can actually be cured by intensive holistic therapy, if they are treated right at onset of disease.

But to work with these "heavy" patients, the therapist must be prepared to invest his whole existence and he must expect to be challenged at all levels of existence. To maximize the therapeutic effect of psychodynamic psychotherapy, it must be turned into the system of CHM, also using bodywork and philosophy of life. CHM has been developed through the last 2 decades to treat even severely mentally ill patients, such as those with schizophrenia.

There are many different factors to get hold of and a lot of theory to master before a therapist can treat schizophrenic patients easily. As a therapist, you need to acknowledge the gift of a daily challenge. You need to appreciate the gift of not getting gratefulness in return for a most demanding and even exhausting job. You need to like to work with the human shadow, looking deep down into the evil, the sexual, the mysterious, and the divine side of life. We need therapists and researchers to engage in the development of CHM because we owe the patient a treatment that cures them, not just a treatment that alleviates some of the symptoms and gives many more side effects. If you can love even the sickest person, if you can see all the illnesses you meet in your patient in yourself too, if you want to develop yourself and obtain wisdom, then working with schizophrenic patients might be the job of your dreams.

We find that psychodynamic treatment/CHM treatment is the cure of choice for schizophrenia, as the psychopharmacological treatment has proved less efficient with many side effects. We therefore recommend publicly funded research in scientific holistic therapy and its development.

## ACKNOWLEDGMENTS

The Danish Quality of Life Survey and the Quality of Life Research Center was 1991–2004 supported by grants from the 1991 Pharmacy Foundation, the Goodwill-fonden, the JL-Foundation, E. Danielsen and Wife's Foundation, Emmerick Meyer's Trust, the Frimodt-Heineken Foundation, the Hede Nielsen Family Foundation, Petrus Andersens Fond, Wholesaler C.P. Frederiksens Study Trust, Else & Mogens Wedell-Wedellsborg's Foundation, and IMK Almene Fond. The research was approved by the Copenhagen Scientific Ethical Committee under number (KF)V.100.2123/91 and further correspondence.
